# Surgery

**Published:** 1913-12

**Authors:** C. Ferrier Walters


### SURGERY.

**Recent Advances in the Treatment of Gonorrhœa.**—Considering the prominence which syphilis and its modern treatment has recently assumed, the importance of efficient and early treatment in the other venereal scourge, gonorrhœa, cannot be too often emphasised. With modern methods persistently applied, there are practically no cases in which a cure is not eventually obtained. It is the dalliance with the chemist and the lay advice, too often followed, wherein lie the most fruitful sources of the chronic stages which try, even the expert, to the utmost to subdue, although the pessimistic utterance of Ricord, that "one knows perfectly well when a clap begins, but that it is the privilege of God of being able to tell when it will end" does not quite hold good to-day.

The methods of accurate localisation of the disease are often neglected, and the varieties of treatment are so many, that one is at a loss as to which is the most efficient and at what stage it should be applied, and falls back on the inevitable injection in and out of season, in the often disappointed hope that all signs and symptoms of the disease will disappear before patient and physician are wearied of the sight of one another.

A brief outline of the recent progress made in the treatment of this fell disease should be of value, for it is possible to lay down more or less definite lines of treatment for the majority

[1] *Bull. et Mém. Soc. de Radiol. Méd. de Paris*, No. 31, 1912.

of cases, although exceptions arise in a small minority, where intolerance and idiosyncrasy prevent their application.

Under favourable conditions a gonorrhœa, as is well known, may terminate in six weeks, and presents during that period three more or less definite stages, viz. that of acute purulent and increasing discharge, with its associated severe discomfort; that of diminution in the amount and thickness of the discharge and gradual disappearance of pain and discomfort; and lastly, that of a thin and gradually subsiding discharge, each stage being roughly a fortnight in duration. The question of aborting the disease should be considered, but it is useless to do so forty-eight hours after its appearance, the gonococci having already penetrated the epithelium, in which a safe refuge is found from all antiseptics of a suitable strength.

Should the patient be sensible enough to go to the right source of treatment at once, the best method of averting the disease is that of irrigation of the anterior urethra by Janet's[1] method, night and morning, with large quantities and increasing strengths of potassium permanganate, from 1–5,000 to 1–1,000, over a period of ten days, when eighty-seven per cent. of cures are obtained if the disease is thus attacked twelve hours after its appearance.

The efficacy of the treatment can then be determined by allowing the patient to resume his ordinary life, including alcohol, and should the discharge reappear, another course of irrigation for ten days should be instituted.

It is, unfortunately, seldom that the opportunity for abortion arises, and even then the irrigation treatment can only be carried out in private practice and at special hospitals. Until the day arrives when the community looks upon those attacked with gonorrhœa, as a result of their own indiscretion, as unfortunate fools only, it is unlikely that the proportion amenable to this treatment will increase.

Usually it falls to one's lot to apply antiphlogistic treatment to the acute stage of gonorrhœa, which, as Luys puts it, "is not destined to cure but to relieve."

Its first object is the removal of all factors which tend to act adversely on the course of the disease.

Rest, general and local, is advisable, and in the latter a suspender which holds the penis in a vertical position on the abdomen is of advantage, as it obliterates the peno-scrotal angle and prevents damming back of the discharge.

The relief obtained by the intake of large quantities of fluid, of a bland nature, is well recognised, and the addition of an alkali to the various urinary sedatives is of value in diminishing the scalding. To direct the patient to pass urine with the

[1] Luys, *Gonorrhœa and its Complications*, Translated and Edited by A. Foerster, 1913, p. 278.

penis immersed in a glass of hot water also relieves this conside ably.

Leeches to the perineum in severe cases are of value, and for painful erection bromide of camphor, in 1-gram doses half an hour before bed-time, is said by Luys [1] to be an improvement on the bromide of potassium.

This treatment should be adopted for about a fortnight, until the pain during micturition ceases, and the second stage is reached, when urethro-vesical irrigations of potassium permanganate, 1–5,000, by Janet's method, should be commenced. The risk of infecting the deep urethra, sometimes feared, is *nil*, and this should be carried out even if no sign of posterior urethritis is present.

They should be continued once or twice daily for two weeks, then on alternate days, every fourth day, and finally once a week, and if no discharge is present after an interval of a week, they should be discontinued.

The strength should be increased if the discharge continues with little diminution, and diminished if pain and scalding are aggravated.

During the second stage the administration of sandal-wood oil is advisable, and the patient should be directed to urinate as frequently as possible, so that the drug in the urine is brought into contact with the urethral mucous membrane as often as possible; but it is only a source of increased irritation if administered in the acute stage.

In the event of posterior urethritis supervening, as indicated by frequency, difficulty of micturition, and, occasionally, by hæmaturia at the end of micturition, hot rectal injections, morphia and urotropine are indicated; but urethro-vesical irrigations should be discontinued until the acute symptoms subside, and then reinstituted with permanganate or 1–3,000 silver nitrate and the prostate massaged.

The presence of chronic post-urethritis is revealed by the three or five glass method, the examination being carried out on the early morning urine, or three or four hours after the previous micturition.

The onset of posterior urethritis during the course of an infection of the anterior urethra alters the prognosis, and one can say with confidence that the advent of cure will be delayed, even with thorough treatment, for a period of some months, and very frequently a year, and unless the patient persists may remain for an indefinite period. Although Keyes [2] has found that the gonococcus, apart from reinfection, rarely survives for more than three years, it is probable that it may remain dormant for much longer. The urethro-vesical irrigations should be associated with dilatation of the posterior

[1] Luys, *Op. cit.*, p. 275. [2] Keyes, *Tr. Am. Urolog. Ass.*, 1911, v. 37.

urethra by Kollmann or other dilators, and massage of the prostate, vesiculæ seminales, and the glands of the urethra against a metal sound is commenced. The occasional parameatal crypts on the glands should also be sought for, and if present examined as a likely hiding-place for the organism, and as a cause of the persistence of the disease.

The value of dilating lies in its action of keeping the mouths of infected glandular ducts and crypts well open, so that the subsequent massage and irrigation may have its full effect.

Kollmann's irrigating dilator is one of the most satisfactory, for its action can be completely controlled, hemorrhage never occurring if used with care, and its effect in preventing stricture and curing it in its early stages undoubted.

Its action on the granular patches, which often maintain a chronic anterior urethritis, is most marked, and combined with irrigations of silver nitrate or permanganate of potassium, is the best method of dealing with this condition.

The urethroscope is an essential for localising and treating chronic lesions in the anterior and posterior urethra, to which applications of increasing strengths of silver nitrate, etc., cautery, or knife, as indicated, may be directly applied.

The posterior urethra, prostate and vesiculæ are most often responsible for the chronic gleet and the recrudescence of discharge so often the despair of the patient. It is these structures that need special attention in the treatment of chronic gonorrhœa.

The exact condition of the posterior urethra can only be investigated and treated satisfactorily by the urethroscope, which reveals many and varied pathological conditions, of the verumontanum and its neighbourhood, as the source of chronic gleet.

The necessity for prostatic massage and stripping of the vesiculæ is usually manifest, on rectal examination, by the presence of induration in one or both prostatic lobes, and of vesiculæ that are enlarged, firm and painful.

In most cases this treatment reduces both to their normal condition, though sometimes a certain amount of fibrous induration remains, despite its thorough application. But this is not inconsistent with a cure.

The glands of Cowper in resistant cases should be palpated, and if infected will be felt as firm bodies, the size of a pea, between the index finger in the rectum and the thumb on the skin of the perineum, just to the outer side of the bulb ; expression of their contents daily aids in the disappearance of gleet, if its source lies therein.

It is a satisfaction to know that modern methods of examination and recognition of the pathological conditions in the posterior urethra, followed by suitable and persistent treatment, seldom fail to cure.

There are, nevertheless, certain conditions, such as vesiculitis, which resist the most thorough treatment, and manifest their persistence by chronic or recurrent discharge and gonorrhœal rheumatism.

When the usual gamut of treatment for these conditions has failed, and enlarged and tender vesiculæ still exist, the method suggested by Fuller,[1] in 1901, of incising and draining the vesiculæ seminales, via the perineum, should be considered. If statistics prove anything, this is a great advance in dealing with such resistant cases. He records many chronic cases, since 1901, treated by himself and others with most gratifying results, when all other means failed. He says that "the indications for this treatment are chiefly chronic, relapsing urethral discharge and gonorrhœal rheumatism with enlarged vesiculæ." The effect of the operation in acute, bed-ridden cases of gonorrhœal rheumatism is said to be marked, the joint pains disappearing in 24 to 36 hours, the swelling by the fourth or fifth day, and the chronic pains in a week or ten days; and the objection to massage of the joints, which gives rise to an increase in the pain, no longer exists after drainage of the vesiculæ. He instances fifteen cases of severe acute rheumatism subjected to this treatment, of which thirteen were promptly cured and two radically relieved; three subacute cases, of which two were cured, and one developed later tuberculous disease in these organs; two relapsing cases cured; seven chronic cases of many months' duration, five promptly, one eventually cured, and one relieved; eight chronic cases of years' duration, four cured and two much relieved; one relieved, but the joints not yet well, and one of eighteen years' duration relieved for six months, followed by relapse.

In 1909 Fuller[2] reported 251 cases on which he had operated without mortality and with uniformly good results; moreover, the excellent effect of this proceeding has been confirmed by other surgeons. One not infrequently meets with the condition known as "pus-tubes in the male," namely, a hard nodule at the lower end of the epididymis appearing after an attack of epididymitis and persisting. This condition is one of the sources of recurrent discharge and chronic gleet, and leads to blocking of the vas of the affected side by fibrous tissue.

Belfield,[3] has opened and drained the vas for this condition, The value of gonococcal vaccines in acute urethritis appears to and for treatment of chronic vesiculitis by irrigation; and the method seems to hold out some promise of more rapid cure.

[1] Fuller, *J. Am. M. Ass.*, 1901, xxxvi. 1228.

[2] Fuller, *Med. Rec.*, 1909, lxxvi. 717.

[3] Belfield, *Tr. Am. Urolog. Ass.*, 1909, iii. 13.

be small, and in chronic cases the results are not particularly encouraging. Not infrequently one is asked when a patient is fit to marry after gonorrhœa. This has, in the past, been a most difficult question to answer.

One knows the difficulty, often experienced, in finding the gonococcus in chronic cases, how that plate culture after plate culture may be made, and no gonococcus found, the patient being eventually told he is fit to marry, and the fallacy of one's advice proven by the development of a gonorrhœal discharge in the wife, or even peritonitis. It is to be hoped that a means has now been found, to prevent the giving of such calamitous advice, in the complement fixation test for gonorrhœa, first applied by Müller and Oppenheim in 1906,[1] to the study of the serum of a patient with gonorrhœal arthritis. Torrey[2] in 1907 confirmed the fact that all anti-gonococcal sera have power to bind complement, and that a particular strain of gonococcus only reacts strongly to serum from an animal immunised with that strain, proving the existence of different strains of this organism.

Schwartz,[3] with this in view, tested 324 human sera from patients with acute or chronic gonorrhœa, and proved the necessity for a polyvalent antigen. In this test he used an antigen prepared from a dozen different strains of gonococcus, and followed the technique of the Wassermann reaction in his experiments with the complement fixation test for gonorrhœa. He found that a positive reaction denotes the presence of or the recent activity of a focus of living gonococci in the body.

Schmidt,[4] Swinburne,[5] Gardner and Clowes,[6] and other workers have tested its value on human sera in many hundreds of cases, and found that no normal individual gives the reaction, and that diseases such as syphilis and tubercle, with definite deviation phenomena, do not react to this test, which therefore has a high degree of specificity. No reaction is obtained for three weeks, coinciding with the period during which the disease is confined to the anterior urethra, and the absorption of toxins

[1] Müller and Oppenheim, *Wien. Klin. Wchnschr.*, 1906, xix. 894.

[2] Torrey, "Agglutinins and Precipitins in Anti-Gonococcic Serum," *J. Med. Research*, 1907–8, xvi. 329.

[3] Schwartz, "The Complement Fixation Test in the Diagnosis of Gonorrhœa," *Am. J. M. Sc.*, 1911, cxli. 693.

[4] Schmidt, "The Complement Fixation Test for Gonorrhœa," *Tr. Am. Urolog. Ass.*, 1911, v. 30.

[4] Schmidt, "Further Report on the Gonorrhœa Complement Fixation Reaction," *Ibid.*, 1912, vi. 370.

[5] Swinburne, "The Complement Fixation Test in Gonorrhœa," *Ibid.*, 1911, v. 21.

[6] Gardner and Clowes, "The Value of Specific Complement Deviation Reaction in Gonorrhœa as an Aid to Clinical Diagnosis," *Ibid.*, 1912, vi. 338.

is slight or *nil.* A marked positive reaction is a fairly certain proof of gonorrhœal inflammation, but a weak reaction, not confirmed by clinical findings, is looked upon as no more positive than a weak Wassermann.

The advantage of such a test is at once recognisable, and with increased knowledge of its possible errors, should be of the greatest value in treatment and prognosis. To be able to say with confidence that a cure is obtained will be a source of great relief to patient and practitioner, and will avoid the disastrous after-effects of the marriage of the still infected. It may have a medico-legal value in that it will be possible to distinguish a recent infection, when a negative, followed in three weeks by a positive reaction, is obtained. Joint troubles, without clinical evidence of gonococcal origin, may be differentiated, and other obscure affections due to the gonococcus elucidated.

C. Ferrier Walters.

